# Digital Innovation and Firm Environmental Performance: The Mediating Role of Supply Chain Management Capabilities

**DOI:** 10.3389/fpsyg.2022.897080

**Published:** 2022-04-28

**Authors:** Mengmeng Wang, Wei Teng

**Affiliations:** College of Business, Gachon University, Seongnam, South Korea

**Keywords:** sustainable growth, digital innovation, supply chain management capability, firm environmental performance, manufacturing industry

## Abstract

Given the omnipresence and profoundness of the ongoing pandemic from the Coronavirus disease 2019, its potential spread can be minimized through social distancing. However, this practice causes increasing difficulties and undesirability of traditional transactions or interactions. Accordingly, various manufacturing firms around the world have become more committed not only to accelerating the development of digital technologies, but also to integrating them with existing processes. In this study, we address an important issue of how manufacturing firms can adapt to the ever-changing volatility and unpredictable global business environment, and achieve sustainable growth by developing a strong supply chain management capability. Two specific interrelated research questions are considered: (1) How do supply chain management capabilities contribute to firm environmental performance; and (2) What are the sources of such capabilities? In so doing, we integrate various forms of digital innovation into a supply chain management capability logic to explore their antecedents and consequences. By using survey data from 272 manufacturing firms in China, we examine the relationship between three key forms of digital innovation (i.e., product, platform, and service) and firm environmental performance. Results show that digital product, platform, and service innovations all have positive contributions to supply chain management capability. In turn, supply chain management capabilities have a partial mediating effect between digital product innovation and firm environmental performance, but a full mediating effect between digital platform and service innovations and firm environmental performance.

## Introduction

Since the beginning of the 21*^st^* century, the rapid development of digital technology has accelerated the reorganization of the supply chain. At present, the digital economy has become the most dynamic and emerging economic and social development, leading future business trends. Considering these developments, major countries in the world have encouraged firms to carry out digital innovation as their main thrust and is regarded as the main kinetic energy to lead their own growth. To comply with the national call and economic environment changes, firms must actively participate and improve in digital innovation, enhance their competitiveness, seize opportunities for future economic development, and gain competitive advantages for performance improvement. Especially in the context of the pandemic from the Coronavirus disease 2019 (COVID-19), face-to-face contact is avoided. Thus, digital platforms are used as important means for major firms to maintain interaction in supply chain nodes. The gap between firms that are leading and lagging in technology has further widened. Digital innovation is used to stabilize risks and turn crises into opportunities, thereby becoming the consensus of all industries. In the post-pandemic era, an industry reshuffle is inevitable. Firms with successful digital innovations are expected to take advantage of the situation and expand in the international market, whereas those who encounter failure would probably withdraw from such arena. Based on its importance, digital innovation has gained the extensive attention of academic circles. In the dimension of digital product innovation, firms recombine digital and physical components to produce new products, use digital resources to produce new artifacts, and apply digital technology to add new attributes and functions to existing non-digital products, thus creating new use values (Nambisan et al., 2017). In the dimension of digital platform innovation, firms use digital tools such as process, project, and information management programs to improve their operational efficiency (Boland et al., 2007). The innovation of digital platform helps firms with the following: improving the existing processes and functions; meeting dynamic business requirements; reducing the time, costs, and resources needed for development and deployment; and identifying digital opportunities to cope with the changes in the internal and external environments (Sedera et al., 2016). In the dimension of digital service innovation, firms use digital technology to communicate with customers in real time, provide timely feedback, and solve customer problems to provide them with completely different solutions and improve their engagement in company efforts (Kim et al., 2021). Thus, with the continuous integration of digital technology into the product, service, and business process transformations, digital innovation can be subdivided into digital product, platform, and service innovations.

With the influence of digital innovation, firms can shorten the R&D cycle, save resources, and realize value creation (von Briel et al., 2018), reduce the time to market of products (Fichman et al., 2014), and use digital technology on a large number of simulation experiments, thereby eliminating the need to repeatedly develop abrasives and saving resources (Vaccaro et al., 2011). In terms of relevant research, finding the shortcomings is not difficult. First, existing research mainly comprises case studies whereas quantitative analyses are relatively few. Second, as digital innovation is a complex concept that covers various levels such as products, platforms, and services, their refinement into these three dimensions is necessary. Finally, although digital innovation provides various benefits, no research has focused on the relationship among digital innovation, supply chain management capability, and firm environmental performance.

The considerable development of global economic integration leads to the increasing commonality of multinational operations. Take the manufacturing industry as an example; for the finished products received by consumers, the product design, raw materials, production, and assembly of components may come from different countries. Before products enter the consumer market, its manufacturing is carried out by a considerable number of firms. Given the different geographical locations, production levels, and management capabilities of these firms, fluctuations in market demand and lack of effective supply chain management may easily lead to the “bullwhip effect”. This scenario magnifies a demand variation in the supply chain, severely affecting their entire value output. In the digital era, firms use digital technology to reduce product development cycles, inventory levels, and delivery time. In addition, through digital innovation, firms provide customized products and services. The pressures of pre-sales, after-sales, and operation costs are maintained in balance to meet the increasing digital demand of consumers and operate to benefit every node in the chain. When companies carry out digital innovations, the constant upgrading of their products is driven by technology. The shortened product life cycle has led to increased fluctuations in product demand. In addition, digital innovation has caused an unprecedented level of requirements for supply chain management capabilities. On the supply side, firms capture demand from new users or even meet potential ones through digital innovation, which puts supply chain management capabilities to the test. Improving supply chain management capabilities enables the relevant firms to share information, resources, benefits, and risks. In addition, all stakeholders, such as sellers, buyers, and consumers, can be integrated into a networked chain structure with the aim to maximize the overall benefits (Castorena et al., 2014). A company’s supply chain management capabilities mainly include information exchange, coordination and operation, integration of activities, and responsiveness. Specifically, compared with competitors, companies in a supply chain can: exchange information more freely and with better quality; coordinate their operations more efficiently and with less costs; better anticipate demand and plan for the future; and respond faster and more effectively to changing customer and supplier demands (Wu et al., 2006). In the post-pandemic era from COVID-19, only by improving their supply chain management capabilities and rapidly mobilizing digital resources to respond quickly to the crisis can firms become stronger and more resilient. With the spread of COVID-19, increased consumer awareness of environmental protection, global warming, and environmental pollution that increases the risk of virus transmission, companies are deeply reflecting on their past production and management methods to determine how they can innovate to win in the unpredictable post-pandemic era. The relationship between digital innovation, supply chain management, and firm environmental performance is not yet clearly understood by the industry and academia. In response to such situation, this study sets one of the research objectives to determine whether digital product, platform, and service innovations can effectively support firms to improve their supply chain management capabilities. The aim is to systematically understand the impact paths and mechanisms these three innovation dimensions on the firm environmental performance. Empirical analysis is then used to verify the mediation role of supply chain management capabilities in the above relationship. Findings can lay the foundation for subsequent research on digital innovation, to help more firms understand the relationship between digital innovation, supply chain management capabilities, and environmental performance. Apart from filling the research gap in this field, this study can provide a reference for government departments and firms to make effective digitalization and environmental protection decisions.

## Theoretical Background and Hypotheses Development

**FIGURE 1:**
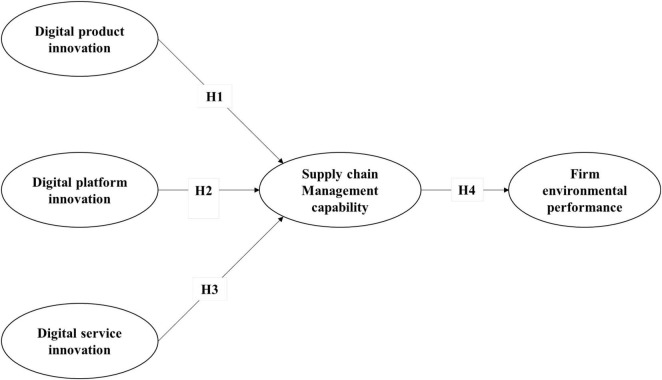
Research model.

Resource-based theory holds that the core competitive advantage of firms is specific resources, which include tangible and intangible ones that can be used in production. Tangible resources are the source of enterprise capabilities. This theory has narrow and broad definitions of resources: the former only regards tangible resources as the key elements of firms whereas the latter considers both tangible and intangible resources (Barney, 2012). According to this theory, an enterprise that has scarce, unrepeatable, lasting, and irreplaceable resources can gain a competitive advantage. The coordination of these resources can improve the enterprise performance and competitiveness (Hart and Dowell, 2011). Digital innovation is an operable resource for firms to gain competitive advantage. In addition, improving the supply chain management ability can optimize enterprise resources (Halldorsson et al., 2007), such as supply chain risk management and learning. Inter-enterprise relationships are also regarded as a type of resource, which emphasizes its importance in mobilizing and integrating the strengths of external partners, and thus bringing the unique advantages of relationships. Referring to the resource-based theory, this study proposes the following theoretical model in Figure 1 and its digital innovation in three dimensions: digital product, platform, and service innovations as independent variables. The mediating variable is supply chain management capability while the dependent variable is firm environmental performance. First, this study discusses the various influences of digital product, platform, and service innovations on the firm environmental performance. Second, as a mediating variable, the role of supply chain management capability is systematically analyzed between the three innovation dimensions and firm environmental performance.

### Digital Product Innovation and Supply Chain Management Capability

Digital product innovation aims to use digital technology to improve existing products, develop new ones, and provide new solutions (Khin and Ho, 2020; Wang, 2021). Enterprise digital technology is regarded as an operational resource and its integration can present different functions (Fichman et al., 2014). Manufacturing firms use digital technology to drive innovation and integrate resources to expand product functions. Digital technology not only enables data to be stored, accessed, and processed, but also feeds back to product development and design to improve environmental performance. Thus, the changing trends of customer demand through data analysis can be effectively predicted to realize accurate product development (Hashem et al., 2015). The application of digital technology in the supply chain can help monitor product manufacturing, predict customer demand, help firms make accurate supply plans, adjust the production schedule as needed, better control the inventory level, reduce inventory and transportation costs, and improve the efficiency of supply chain management (Kwon et al., 2014). Thus, the digital product innovation of firms can have a positive role in improving supply chain management capability. On this basis, the following hypothesis is proposed:

*Hypothesis 1(H1)*: A firm’s digital product innovation is positively associated with the development of the firm’s supply chain management capability.

### Digital Platform Innovation and Supply Chain Management Capability

Digital platforms are external programs based on software, which comprises an extensible code base. Companies and users share information resources on the applications they build (Ghazawneh and Henfridsson, 2015). Digital platforms are widely used in mobile technology, cloud computing, memory technology, and social media, enabling revolutionary changes to the economy and society (Hofmann and Woods, 2010). Firms use digital platforms to build an ecosystem for manufacturers and suppliers, break the boundaries of traditional firms informatization, and reduce the coordination and transaction costs among partners (Harris et al., 2012). By establishing a digital platform with its partners, manufacturing firms share information resources, monitor every node at any time, and improve the entire supply chain (Maroofi et al., 2017). Thus, the digital platform innovation of firms can help improve supply chain management ability. On this basis, the following hypothesis is proposed:

*Hypothesis 2(H2)*: A firm’s digital platform innovation is positively associated with the development of the firm’s supply chain management capability.

### Digital Service Innovation and Supply Chain Management Ability

Digital service innovation aims to illustrate the technological features (Kohtamäki et al., 2019) and can enhance the communication between firms and customers, thereby exerting a positive effect on consumer satisfaction (Khin and Ho, 2020). Digital technology is used in customer-oriented innovation to solve customer problems, technology-oriented innovation to launch new services, and collaboration-oriented innovation to improve the customer relationship and experience, as well as allow their participation in firms operation (Kim et al., 2021). Using digital technology, firms can collect and analyze customer data in real time, delve deep into the potential demands of customers, and increase their awareness of customer needs (Nambisan et al., 2017). Digital technology integrates the resources in the field of service innovation, expands the scope of business, and improves the efficiency of supply chain (Parry et al., 2016). Thus, the digital service innovation of firms plays a positive role in improving supply chain management ability. On this basis, the following hypothesis is proposed:

*Hypothesis 3(H3)*: A firm’s digital service innovation is positively associated with the development of the firm’s supply chain management capability.

### Supply Chain Management Capability and Firm Environmental Performance

The purpose of supply chain management is to maximize the value of products and services by fully mobilizing all of its resources and anticipating customer demand in a timely and accurate manner through information sharing and strategic collaboration (Autry and Griffis, 2008). Supply chain management is a set of coordinated operations from procurement to production all the way to delivery to the consumer. The effectiveness of this process helps companies make informed decisions and minimize the costs of information delays or poor flow (Maroofi et al., 2017). Supply chain management has seven dimensions, namely, strategic supplier partnerships, information sharing level, information quality, customer service management, internal lean practices, delays, and total quality management. Among these dimensions, information sharing and quality have the largest impacts on business performance (Al-Shboul et al., 2017). The sharing of information resources allows the exchange of knowledge among related firms while that of risks allows manufacturing firms to significantly improve their performance (Liu et al., 2013). Supply chain management capabilities have a positive impact on firms performance (Podsakoff et al., 2003). As an important part of firm performance is environmental, which refers to the effectiveness of environmental protection and pollution control in business activities, and the ability to reduce harmful emissions through the supply chain (Dubey et al., 2015). This aspect is mainly expressed in the reduced emissions of hazardous substances, gases, wastewater, and solid wastes; saved energy consumption; and improved environmental awareness (Chien, 2014). The ability of companies to enhance collaboration with suppliers and customers (Siagian and Tarigan, 2021) and manage their suppliers in the supply chain can significantly improve their environmental performance (Dubey et al., 2015). As such, the improvement of supply chain management capabilities can positively contribute to the firm environmental performance. On this basis, the following hypothesis is proposed:

*Hypothesis 4(H4)*: A firm’s supply chain management capability is positively associated with the firm’s environmental performance.

## Methodology

### Sampling and Data Collection

To empirically examine our hypotheses, we collected data from a sample of firms in China’s manufacturing sector. We believe China provides an appropriate research context to examine the effect of a firm’s digital innovation on the firm’s supply chain management capability, which, in turn, contributes to its environmental performance. China, the world’s second largest economy, has become one of the world’s most innovative economies and most important global innovation leaders by undergoing rapid digital transformation. According to the 2021 Global Innovation Index (GII) released by the World Intellectual property Organization (WIPO), China was knocking on the GII top 10’s door by ranking 12th among more than 130 economies in the 2021 GII list. In fact, China was the only middle-income economy among the world’s top 30 most innovative economies. It has been argued that the ongoing innovative design and use of digital technologies in transforming and advancing China’s manufacturing capabilities and developing new business models has been one of the important forces driving its innovation and economic growth (Li et al., 2022). A recent survey report released by global consultancy Accenture shows that many Chinese firms are accelerating their digital transformation to develop sustainable competitive advantages and achieve sustainable growth through the innovative use of new digital technologies such as cloud computing and big data. For example, it was reported that the Digital Transformation Champions accounting for 16% of the surveyed firms generated more than half of their revenues from new businesses over the past three years. More importantly, the report further found that Chinese firms which had digital advantages achieve revenue growth 3.7 times than that of other peers in 2020.

To collect survey data used for the study, we carefully developed the survey instrument by using a double-translation procedure to translate the English survey instrument to Chinese. In doing so, we first developed an English-language version of the questionnaire by conducting extensive literature review and incorporating feedback from four academics. Then, two independent bilingual translation helped us translated it into Chinese. Finally, two different independent bilingual translators back translated the Chinese-language version of the questionnaire into English again to ensure conceptual translation equivalence and accuracy. Prior research (Hoskisson et al., 2000) has suggested the potential challenges faced in collecting useful and sufficient primary data from firms in China and argued for the particular importance of building trust and a good *guanxi* (relationship/personal tie) to obtain high-quality responses in the Chinese market. Therefore, we hired a renowned research institute in the Chinese local market to help us conduct the survey procedures and administer the surveys. Through such survey procedures, we received a total of 281 questionnaires. After eliminating 9 incomplete responses, we received a total of 227 completed and usable questionnaires and utilized them in our final data analysis.

As non-response bias is likely to occur in our sample of firms and thus may influence the interpretation of our empirical results, we assessed the possible presence of non-response bias by comparing the differences between the responding firms and non-responding firms as well as the early-responding firms and late-responding firms, and the results of the comparison revealed that there were no statistically significant differences between these groups in terms of key firm characteristics (e.g., firm size). In addition, like all survey research, our data may also suffer from serious common method variance (CMV). However, we believe that CMV was less likely to occur in our study due to the following reasons. First, we carefully developed the survey questionnaire by keeping it relatively short. Meanwhile, we designed the questionnaire by placing the dependent and independent variables into several subsections with different response format. To reduce the potential concern stemming from social desirability bias, we ensured the respondents that there were no right or wrong answers to the questions included in the questionnaire and that they should answer the survey question from the current perspective of a group of managers rather than from their own. We further encouraged the respondents to participate in the survey by ensuring both the anonymity and confidentiality of their responses in the cover letter accompanying the questionnaire and promising to offer them a summary and evaluation of the findings upon completion of the study, if requested. Nevertheless, we checked for the presence of potential CMV in our data by following Podsakoff et al.’s (2003) recommendation and performing Harman’s one-factor analysis. More specifically, we performed exploratory factor analysis by entering all multiple-item scales into non-rated factor analysis and the results of the one-factor analysis demonstrated that there was no general factor which is apparent in the unrotated factor structure and accounts for a majority of the variance. This finding suggests that our data and results are less likely to suffer from serious CMV problem.

### Variables and Measurement

Unless noted otherwise, we measured all the dependent and independent variables using multiple-item, seven-point Likert scales ranging from “strongly disagree” (1) to “strongly agree” (7).

In this study, to capture a firm’s environmental performance, we asked the firm to assess its overall environmental performance and measured the variable using six items derived from prior research (e.g., Dragomir, 2018; Gölgeci et al., 2019; Weidner et al., 2021; Zhang et al., 2021). Following prior studies (e.g., Khin and Ho, 2020; Wang, 2021), we asked a firm to evaluate its product innovation efforts by adopting digital technologies and measured the firm’s such digital product innovation using eight items derived from prior literature. To measure a firm’s digital platform innovation, we used nine times which were adopted from prior research (e.g., Sedera et al., 2016). Similarly, following prior research (e.g., Woo et al., 2021), we adopted nine items to measure a firm’s digital service innovation. To measure a firm’s supply chain management capability, we carefully reviewed the related literature and adopted a nineteen-item scale to measure the firm’s supply chain management capability (Wu et al., 2006; Peng et al., 2016).

In addition, to control for alternative explanations for the results, we included several variables in the analysis: firm size, industry type, and ownership structure. We measured firm size the annual sales of a firm (Qian et al., 2010; Zhao and Murrell, 2016). To control for the industry effect, we created a dummy variable equal to 1 if the firm is primarily operating in the industrial markets (Takata, 2016). To measure the effect of a firm’s ownership structure, we created a dummy variable equal to 1 if the firm is privately owned (Houston et al., 2011; He et al., 2013).

## Analyses and Results

### Measure Reliability and Validity Assessment

In this study, we used a structural equation modeling (SEM) approach to test the proposed model (Figure 1). Before testing the proposed model and research hypotheses, we first assessed the reliability and validity of the constructs. We summarize the results of the reliability and validity assessment in Table 1. To assess the measure reliability, we used Cronbach’s alpha which has been considered a widely used measure of reliability (Nunnally, 1978). As shown in Table 1, the alpha values of all scales, ranging from 0.908 to 0.976, are greater than 0.70, demonstrating an adequate level of reliability for the measures of constructs used in this study (Nunnally, 1978). To assess the convergent and discriminant validity of the measures, we created a measurement model by conducting confirmative factor analysis (CFA). The fit indexes of the CFA analysis show that the overall model offers satisfactory fit to the data [χ2/df = 1.67, *p* < 0.001; comparative fit index (CFI) = 0.928; Tucker Lewis Index (TLI) = 0.925; incremental fit index (IFI) = 0.929; root mean square error of approximation (RMSEA) = 0.050]. To further check the reliabilities for the constructs, we calculated the composite reliability of each construct and the results shown in Table 1 demonstrated that all the composite reliabilities, ranging from 0.908 to 0.976, are above the 0.70 benchmark, again exhibiting strong internal reliability for our measures. Further, the factor loadings of all indicators are highly statistically significant with values greater than the 0.70 benchmark. In addition, we also calculated the average variance extracted (AVE) statistics, which, ranging from 0.541 to 0.696, are all above the recommended threshold of 0.50. These results provide adequate convergent validity (Fornell and Larcker, 1981). Following Fornell and Larcker (1981), we assessed discriminant validity of the measures by checking whether the square root of AVE of each construct is larger than the correlation between the construct and all possible pairs of other constructs in the model. As shown in Table 2, the results confirmed that the square root of AVE value of each construct is much larger than its correlation coefficients with the other constructs, providing strong evidence for adequate discriminant validity of the measures. Overall, the assessment of the measurement reliability and validity indicates that each construct and their respective indicators exhibit an adequate level of reliability and validity in the context of this study.

**TABLE 1:** Results of reliability and validity assessments of the constructs.

Construct and indicators	Cronbach’s alpha	SFL	CR	AVE
**Digital product innovation (DTI)**	0.908		0.908	0.553
DTI1		0.714		
DTI 2		0.763		
DTI3		0.796		
DTI4		0.774		
DTI5		0.710		
DTI6		0.711		
DTI7		0.761		
DTI8		0.714		
**Digital platform innovation (DMI)**	0.928		0.929	0.591
DMI1		0.746		
DMI2		0.770		
DMI3		0.740		
DMI4		0.750		
DMI5		0.743		
DMI6		0.774		
DMI7		0.819		
DMI8		0.781		
DMI 9		0.793		
**Digital service innovation (DSI)**	0.913		0.914	0.541
DSI1		0.759		
DSI2		0.743		
DSI3		0.751		
DSI4		0.723		
DSI5		0.718		
DSI6		0.728		
DSI7		0.665		
DSI8		0.802		
DSI9		0.724		
**SCM capability**	0.976		0.976	0.686
SCM1		0.798		
SCM2		0.854		
SCM3		0.838		
SCM4		0.825		
SCM5		0.835		
SCM6		0.828		
SCM7		0.796		
SCM8		0.800		
SCM9		0.821		
SCM10		0.813		
SCM11		0.829		
SCM12		0.804		
SCM13		0.835		
SCM14		0.832		
SCM15		0.875		
SCM16		0.819		
SCM17		0.834		
SCM18		0.851		
SCM19		0.842		
**Environmental performance (EP)**	0.931		0.932	0.696
EP1		0.786		
EP2		0.839		
EP3		0.849		
EP4		0.830		
EP5		0.857		
EP6		0.844		

*N = 272. Model Summary: χ2/df = 1.67, p < 0.001, CFI = 0.928, TLI = 0.925, IFI = 0.929, RMSEA = 0.050. AVE = average variance extracted, SFL = standardized factor loading, CR = composite reliability, SCM = supply chain management.*

**TABLE 2:** Descriptive statistics and correlations.

Variable	Mean	STD	1	2	3	5	4	6	7	8
1. Firm size	3.282	1.431	1.000							
2. Industry category	0.608	0.489	0.362**	1.000						
3. Ownership structure	0.620	0.486	−0.162**	–0.052	1.000					
4. Digital product innovation	6.107	0.987	0.080	0.011	–0.024	**0.744**				
5. Digital platform innovation	6.239	0.950	0.099	0.033	–0.028	0.651**	**0.769**			
6. Digital service innovation	6.217	1.028	0.181**	0.134*	–0.036	0.438**	0.486**	**0.736**		
7. Supply chain management capability	2.011	1.137	–0.064	0.009	0.077	−0.615**	−0.392**	−0.386**	**0.828**	
8. Environmental performance	5.993	1.122	0.141**	0.122*	–0.032	0.605**	0.612**	0.543**	−0.546**	**0.834**

N = 272. *p < 0.05, **p < 0.01. Figures reported in bold on the diagonal are the square root of the average variance extracted for the constructs.

### Hypotheses Testing

**FIGURE 2:**
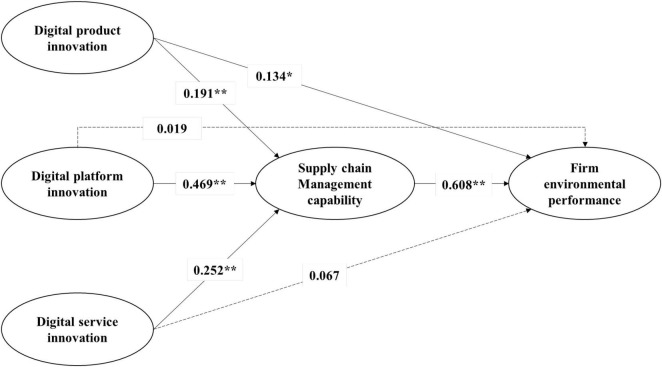
Estimated results of the hypothesis tests using a structural equation modeling. Note: Model summary: χ^2^/df = 1.67, *p* < 0.001, CFI = 0.928, TLI = 0.925, IFI = 0.929, RMSEA = 0.050. Non-significant paths are shown by a dotted line. *p < 0. 05 and **p < 0.01.

Following the measurement model assessment, we empirically examine the hypotheses by performing structural equation modeling. We present the results of SEM analysis in Figure 2. We assessed the overall structural model fit and all indexes of model fit demonstrate that the sample data fit the hypothesized structural model reasonably [χ2/df = 1.67, *p* < 0.001; comparative fit index (CFI) = 0.928; Tucker Lewis Index (TLI) = 0.925; incremental fit index (IFI) = 0.929; root mean square error of approximation (RMSEA) = 0.050]. Overall, the results presented in Figure 2 indicate that the constructs are largely related in the theoretically predicted manner. More specifically, the results show a significant positive relationship between all the three digital innovation variables, i.e., digital product innovation (ß = 0.191, *p* < 0.01), digital platform innovation (ß = 0.469, *p* < 0.01), digital service innovation (ß = 0.252, *p* < 0.01), and the development of supply chain management capability. Therefore, these results indicate that a firm’s digital product innovation, digital platform innovation, and digital service innovation, as hypothesized, are key determinants of the development of the firm’s supply chain management capability. These results thus provide strong support for Hypotheses 1, 2, and 3.

Furthermore, we tested Hypothesis 4 by examining the possible role of a firm’s supply chain management capability in predicting the firm’s sustainable environmental performance. As reported in Figure 2, the path coefficient between the supply chain management capability and environmental performance is positive and statistically significant (ß = 0.608, *p* < 0.01). This result indicates that a firm which has a stronger supply chain management capacity is expected to achieve a better environmental performance. On the basis of this empirical evidence, Hypothesis 4 is also strongly supported.

### Supplementary Analysis

We examine the possible mediating effect of supply chain management capability in the relationships between the three digital innovation variables and firm environmental performance. Although exploring this possible mediating effect goes beyond the scope of this study, we empirically investigate such possibilities as a supplementary analysis. Following Zhao et al. (2010), we use empirical tests to verify the potential mediating effect of supply chain management capability by determining their significance. Consistent with our expectations, the results shown in Figure 2 provide strong evidence that a firm’s supply chain management capability fully mediates the relationship between its digital platform innovation and its environmental performance. In this relationship, the coefficient of the indirect effect is positive and statistically significant (ab: ß = 0.285, *p* < 0.01) but that of the direct effect is not statistically significant (c: ß = 0.019, n.s.). Similarly, the results also suggest that a firm’s digital service innovation has a positive and statistically significant indirect effect on the firm’s environmental performance, which is fully mediated by the firm’s supply chain management capability. In this relationship, the coefficient of the indirect effect is positive and statistically significant (ab: ß = 0.153, *p* < 0.05) but the direct effect is not statistically significant (c: ß = 0.067, n.s.). Overall, the results in Figure 2 provide evidence of full meditating effects of supply chain management capability in the relationships of digital platform and service innovations with environmental performance (Zhao et al., 2010). In terms of the effect of digital product innovation on environmental performance, the results reveal that not only is the direct effect positive and statistically significant (c: ß = 0.134, *p* < 0.05), but also the indirect effect is positive and statistically significant (ab: ß = 0.116, *p* < 0.01). These results imply a presence of complementary or partial mediating effect of supply chain management capability in the relationship of digital product innovation with environmental performance (Zhao et al., 2010). In the following section, we discuss these results and their implications.

## Discussion and Conclusion

The COVID-19 pandemic has accelerated the importance of digital platforms as a means for firms to maintain interaction in their supply chains. Thus, digital innovation has become a strategic firm choice, and the gap between firms that are leading and lagging in technology has further widened in terms of competitiveness. To help firms grasp the development initiative in the post-pandemic era, we need a clear understanding of the relationship among digital innovation, supply chain management capabilities, and firm environmental performance. By using the resource-based theory, this study divides the digital innovation strategy into digital product, platform, and service innovations, and discusses their various influences on supply chain management capabilities. Using unique survey data collected on a sample of manufacturing firms in China, we provide and discuss the following findings. First, supply chain management capabilities have a partial mediating effect between digital product innovation and environmental performance. Companies can use digital technology to innovate their products to achieve a double effect, namely, improve their supply chain management capabilities, which can also directly improve their environmental performance. Companies that cannot build digital platforms or provide digital services can instead prioritize the implementation of digital product innovations according to their own situation. Moreover, supply chain management capabilities have a full mediating effect between digital platform innovation and environmental performance. Among the three major digital innovations, that of platforms has the greatest impact on supply chain management capabilities, that is, a positive impact that is not directly related to environmental performance. Digital platform innovation can only indirectly improve environmental performance through supply chain management capabilities, which firms are thus recommended to enhance when building digital platforms. Finally, supply chain management capabilities have a full mediating effect between digital service innovation and environmental performance. In other words, digital service innovation can indirectly but not directly improve firm environmental performance by enhancing supply chain management capabilities. These empirical findings provide theoretical basis for firms to implement strategies in the post-pandemic era and help them determine how to implement digital innovation to improve supply chain management capabilities and firm environmental performance. In this study, digital product, platform, and service innovations are set as independent variables, with supply chain management capability as a mediating variable and firm environmental performance as a dependent variable. A survey of 272 firms reveals the relationship among the three dimensions of digital innovations, supply chain management capability, and firm environmental performance. A comprehensive model is developed and designed to provide useful reference for firms to implement digital innovation strategy in the post-pandemic era, promote and improve firms supply chain management, and provide effective solutions for maximizing firm environmental performance.

Through empirical analysis, our study contributes to the literature in the following ways.

First, previous literature emphasized that digital product innovation can create new use value for customers (Nambisan et al., 2017). The present study finds that digital product innovation has a clear promoting effect on supply chain management ability. Thus, firms can use digital technology to add new attributes and functions to existing products. In addition, digital technology can be applied to development, design, and marketing links to enable faster and more effective responses to the ever-changing needs of customers and suppliers, as well as to allocate according to demand, and better control the inventory to rapidly improve the supply chain management capability.

Second, previous literature emphasized that digital platform innovation can help improve the operational efficiency of firms (Boland et al., 2007), breaking the boundaries of traditional informatization and reducing the coordination and transaction costs among partners (Harris et al., 2012). Moreover, the dynamic business requirements can be fulfilled with reduced time, costs, and resources for firms development and deployment (Sedera et al., 2016). Consistent with previous literature, this study proves that digital platform innovation has a positive effect on supply chain management capability. Firms are suggested to actively use digital tools such as process, project, and information management programs to strengthen the contact with suppliers and customers, and thus fulfill their changing needs. Build digital platforms with partners and customers, share information resources, and improve their abilities in activity integration, information exchange, and coordination response in the supply chain such that all partners can maximize their interests.

Third, in previous literature, the innovation of digital services enhances the communication between firms and customers (Khin and Ho, 2020) to improve the latter’s participation (Kim et al., 2021). The present study finds that digital service innovation has a positive effect on firms supply chain management capability. Firms are encouraged to actively use digital technology to carry out service innovation when solving customer and partner problems, provide real-time communication and quick feedback of digital services for cooperation activities, improve the coordination response and information exchange ability in supply chain management, and work together with suppliers to plan future demand.

Lastly, in general, supply chain management capabilities have a positive effect on firm performance (Podsakoff et al., 2003; Hsu et al., 2009), of which environmental performance is important. This study extends previous findings to verify that improving supply chain management capabilities can significantly improve firm environmental performance. The results demonstrate a partial mediating effect of supply chain management capability in the relationship between digital product innovation and firm environmental performance. Therefore, a firm may choose to prioritize the implementation of digital product innovation if they have no sufficient digital platform or service innovation. Furthermore, the results also show that supply chain management capability has a full mediating effect in the relationship between digital platform innovation and firm environmental performance. More importantly, among the three types of digital innovation, digital platform innovation has the greatest impact on supply chain management capabilities, indicating its important positive influence on the development of supply chain management capabilities but no significant direct effect on firm environmental performance. Given this indirect effect, firms can rather focus on developing and enhancing their supply chain management capabilities when attempting digital platform innovation. Moreover, the results suggest that supply chain management capability also fully mediates the effect of digital service innovation on firm environmental performance. In other words, digital service innovation can only exert an indirect effect by enhancing the supply chain management capabilities but not directly improve environmental performance. Overall, building on resource-based theory, this study offers important contributions to relevant literature by delving deeper into the mediating effect of supply chain management in the relationships between specific types of firm digital innovation and environmental performance, and providing empirical evidence for one of the three mechanisms. Therefore, we hope that this study can enrich the literature on resource-based theory and provide meaningful, practical guidelines for managing supply chains and digital innovation strategies.

### Limitations and Future Research Directions

Similar to any research, this study faces certain limitations. First, given the influence of time, energy, and economy, we only investigate Chinese firms. The varying digital innovations and supply chain management capabilities in various countries and firms necessitates the further expansion of this research methodology to greatly improve its universality. Second, compared with that for individual users, the use of survey for firm users is difficult and yields a low feedback rate. During the survey, many firms are reluctant to disclose sensitive information, such as their financial performance. Thus, the firm environmental performance can only be studied through scales. In the future, various channels can be used to obtain relevant information and maximize the research persuasiveness by comparing second-hand and original firm data. Third, in this study, we only consider the mediating role of supply chain management capability in the relationship of digital innovation and firm environmental performance. However, we believe that the contribution of digital innovation to environmental performance may be mediated or even moderated by other organizational and environmental factors. Therefore, future research is encouraged to explore these factors, such as digital technology capabilities, leadership, and supply chain risks, that may mediate or moderate the relationship between digital innovation and environmental performance. We hope an extension of this study can provide more new and useful insights about how to further benefit from implementing digital innovation strategies. Finally, more valuable suggestions can be provided for the implementation of firms’ digital innovation strategies. Fourth, regarding supply chain management capabilities, this study focuses on core upstream and downstream firms because of the possible involvement of different partners at various levels, which increases its complexity. Another direction of improvement in future research is to examine more specific issues at different upstream and downstream stages. Finally, while the results of our study provide evidence for full or partial mediating roles of supply chain management capability in the relationships between specific types of digital innovation and environmental performance, several important issues remain. For example, what are the plausible mechanisms that firms can use to better transform their digital innovations into performance? Additional studies thus need to further unpack the specific mechanisms underlying different mediating effects of supply chain management and other firm-specific capabilities. Such research attempts are of significant theoretical and practical importance to more deeply understand the role of digital innovations on firm performance.

## Data Availability Statement

The raw data supporting the conclusions of this article will be made available by the authors, without undue reservation.

## Author Contributions

Both authors listed have made substantial, direct, and intellectual contributions to the work, and approved it for publication.

## Conflict of Interest

The authors declare that the research was conducted in the absence of any commercial or financial relationships that could be construed as a potential conflict of interest.

## Publisher’s Note

All claims expressed in this article are solely those of the authors and do not necessarily represent those of their affiliated organizations, or those of the publisher, the editors and the reviewers. Any product that may be evaluated in this article, or claim that may be made by its manufacturer, is not guaranteed or endorsed by the publisher.
